# Validation of the personal suicide stigma questionnaire among adolescents with suicide attempts in mainland China

**DOI:** 10.3389/fpsyt.2024.1445247

**Published:** 2024-09-13

**Authors:** Xiaoning Wang, Qunfang Miao, Jiannv Wang, Lingjing Qiu, Jinsheng Zhang, Peiqing Li, Yaoyao Huang

**Affiliations:** ^1^ School of Nursing, Hangzhou Normal University, Hangzhou, China; ^2^ Clinical Medicine, Hangzhou Normal University, Hangzhou, China; ^3^ Nursing Department Hangzhou Seventh People’s Hospital, Hangzhou, China

**Keywords:** adolescents, suicide attempts, Chinese version, reliability, validity, personal suicide stigma questionnaire

## Abstract

**Objectives:**

The study aimed to translate and culturally adapt the personal suicide stigma questionnaire (PSSQ) into simplified Chinese and evaluate its psychometric properties among adolescents who have attempted suicide in mainland China.

**Methods:**

Following Brislin’s translation model and using purposive sampling, we surveyed 440 adolescents who had attempted suicide at Hangzhou Seventh People’s Hospital in Zhejiang Province, China. Content validity was determined by a panel of experts, and the construct validity of the scale was assessed using exploratory factor analysis (EFA), confirmatory factor analysis (CFA), convergent validity, and discriminant validity. Reliability analysis was evaluated using Cronbach’s α coefficient, test–retest reliability, and half-split reliability.

**Results:**

The Chinese version of the PSSQ consists of three dimensions and 14 items. After two rounds of expert consultation, the item-content validity index for all items exceeded 0.70, and the scale-content validity index exceeded 0.90. EFA extracted three factors and retained all 14 items. The CFA indicators demonstrated a good fit. The Cronbach’s α coefficient of the scale was 0.880, the half-split reliability was 0.681, and the test–retest reliability was 0.862. It is evident that the PSSQ and its subscales demonstrate stable structural validity and good internal consistency in measuring self-stigma among individuals with suicidal tendencies, indicating that the PSSQ is a reliable tool for assessing the degree of personal stigma in Chinese adolescents who have attempted suicide.

**Conclusion:**

This study ensured the linguistic and cultural appropriateness of the Chinese version of the PSSQ through cross-cultural adaptation and validation of its reliability and validity, thereby enhancing the accuracy and reliability of assessing personal stigma among Chinese adolescents who have attempted suicide. The validation of the Chinese version of the scale not only enriches the research tools available for studying personal stigma related to suicide in mainland China, but also provides a reliable quantitative tool for future research on the psychological states of individuals who have attempted suicide, the impact of stigma, and the effectiveness of interventions.

## Introduction

In the context of rapid societal development, suicide has increasingly become a prominent, sensitive, and serious social challenge. Adolescents, as a distinct social group, are a focal point of academic attention due to their high incidence of suicide attempts. A suicide attempt refers to an individual’s intention to end their own life that does not result in death (1). Research has indicated that each person who dies by suicide has between 10 and 40 nonfatal suicide attempts before completing suicide (2). In recent years, reports of suicide attempts among Chinese adolescents have become increasingly frequent. In a comprehensive analysis of cross-sectional studies on suicide attempts among Chinese adolescents conducted by scholar Hu and colleagues (3), it was found that the overall prevalence of suicide attempts in this population is approximately 2.94%. Specifically, the prevalence is 2.5% for male and 3.17% for female adolescents. The escalating severity of adolescent suicide can be attributed to the incomplete psychological development during puberty, combined with substantial pressures from family, society, and school (4). These factors make adolescents more vulnerable to negative emotions and potential suicidal behavior. Suicide attempts not only cause severe physical pain for adolescents but also subject them to stigma and judgment from others (5). Stigma represents a negative stereotype that can have severe adverse effects on the physical and mental health, as well as academic and social functioning, of those who are stigmatized. Juliane Brüdern’s research indicates (6) that discriminatory and indifferent attitudes toward individuals who attempt suicide elevate the risk of repeated suicide attempts among adolescents. Simultaneously, adolescents may internalize the stigma imposed by others, leading to self-stigmatization and further questioning of their own value of life. Rimkeviciene and colleagues (7), in their qualitative study, identified that suicide attempts result in negative personal stigma experiences for adolescents, such as feelings of shame, labeling, and discrimination.

Simultaneously, individuals who attempt suicide represent a high-risk group for subsequent suicides, placing a significant burden on both families and society. Some researchers suggest that the transition from attempted suicide to subsequent suicides represents a continuous process (8). A survival tree analysis conducted in France reveals that the initial 6 months following a suicide attempt constitute a high-risk period for subsequent suicides. Among those who have attempted suicide, 1.6% die by suicide within the next 12 months and 3.9% die by suicide within the following 5 years (9).

Scholars have suggested that the stigma associated with suicide experienced by individuals following a suicide attempt, particularly among adolescents, may contribute to an increased risk of subsequent suicidal behavior (10, 11). In a qualitative synthesis study, Carpiniello and colleagues explored the relationship between suicide stigma and suicidal ideation, indicating that while suicide often results in stigma, this stigma may, in turn, contribute to an increased risk of subsequent suicides. To prevent such recidivism, efforts should be intensified to combat the stigma associated with suicide itself (10). In a qualitative study by Oexle and colleagues, on stigma associated with suicide attempts and suicidal ideation, it was demonstrated that the stigma experienced by individuals following a suicide attempt increases their risk of subsequent suicide (12). The study identified themes such as loneliness and despair as significant precursors to repeated suicidal behavior (12). Thus, it is evident that suicide stigma is a significant risk factor for suicide. Reducing suicide stigma will be crucial for preventing repeat suicides among adolescents.

However, to effectively reduce suicide stigma, patients must first disclose their suicide attempts. Disclosure is the first step in prevention and intervention and is also crucial for improving individuals’ emotional states and behaviors. Adolescents who have attempted suicide may feel ashamed to openly discuss their experiences, which can interfere with their willingness to seek help and increase their suffering (13). A study on suicide stigma found that perceived stigma associated with suicide, combined with subsequent concealment of suicidal behaviors, contributes to the deterioration of mental health in individuals who have attempted suicide (14). Adolescents are at a critical stage for information development, yet their cognitive levels and abilities are still underdeveloped. As a result, they may struggle to accurately analyze information and concepts related to suicide, leading to lower suicide literacy (15). Furthermore, the concealment of suicide attempts and reluctance to seek help proactively can further internalize the stigma associated with suicide.

Qualitative research in China has indicated that adolescents who have attempted suicide experience strong feelings of shame regarding their suicidal behavior and have thoughts of further self-harm (16). Personal stigma arising from a suicide attempt can impede the psychological and physical recovery of adolescents (17). It is necessary to accurately assess the personal stigma of adolescents who have attempted suicide to reduce the occurrence of further suicidal behavior resulting from the personal stigma associated with a suicide attempt. However, there has been no quantitative research on personal stigma related to suicide in China, and there are currently no assessment tools specifically designed or introduced for the personal stigma associated with suicide. The personal suicide stigma questionnaire (PSSQ), developed by Australian scholar Jurgita and colleagues (18), in 2019, was primarily used to measure the level of personal stigma in suicide attempters. It has comprehensive content and demonstrates good reliability and validity. Currently, there is no Chinese version or versions in other languages available. Consequently, this study aimed to adapt and validate the PSSQ across cultures, establishing a Chinese version of the PSSQ suitable for the Chinese context. This will facilitate a precise evaluation of the current personal suicide stigma among adolescents who have attempted suicide in China, offer a basis for developing targeted intervention strategies, and enhance the body of research on personal suicide stigma conducted by Chinese scholars.

## Methods

### Ethical considerations

This study was approved by the Ethics Review Committees of the School of Nursing, Hangzhou Normal University (2023110), and the Seventh People’s Hospital of Hangzhou (2023058). The survey strictly adhered to the principles of informed consent, confidentiality, and non-injury. Before the survey, the purpose and significance of the study were thoroughly explained to ensure that patients could make an independent decision on whether to participate after fully understanding the research content. We promised that the relevant information of the research subjects would be kept strictly confidential and used only for research purposes.

### Participants

From October 2023 to January 2024, a purposive sampling method was used to select adolescent patients with a history of suicide attempts admitted to the inpatient department of Hangzhou Seventh People’s Hospital in Zhejiang Province, China, as the research subjects. The inclusion criteria were as follows: (1) age between 10 and 19 years (as defined by the World Health Organization for adolescents), (2) meeting the definition of a suicide attempt, and (3) having experienced at least one suicide attempt. The exclusion criteria were as follows: participants with severe mental illness or cognitive impairment were unable to participate in the study. Based on the recommendation of 5–10 times the number of items in the scale, that is, sample size = (number of scale items) × (5–10) (19, 20), and considering a 20% invalid sample rate, the minimum sample size required for this study was calculated to be 96, with a maximum sample size of 192. However, considering that a minimum sample size of 200 cases was required for confirmatory factor analysis (CFA), the data for exploratory factor analysis (EFA) and CFA were not replicable; it was estimated that at least 300 cases were needed. This study surveyed 445 cases. A quantitative research approach was employed to develop and validate the scale, as well as to assess its reliability and validity. The choice of a quantitative method was based on its advantages in ensuring the objectivity and generalizability of the research findings. Through meticulous questionnaire design and the use of advanced statistical analysis tools, this study conducted a comprehensive reliability and validity analysis on a large-scale sample to ensure that the scale possesses robust psychometric properties.

The survey tools included a general information questionnaire and the Chinese version of the Suicide Stigma Pretest Scale. The general information questionnaire was developed by the researchers based on the content and purpose of this study after reviewing domestic and foreign literature. It included gender, age, residence, school enrollment status, only child status, primary caregiver, parents’ marital status, family history of mental illness, family history of suicide, doctor’s diagnosis results, frequency of suicide, reasons, methods, the most recent suicide attempt, and attitude toward death. There were no patients who refused to participate in the study during the research process.

### Instruments

The PSSQ was developed by Jurgita and colleagues (18). in Australia in 2019. The scale consisted of three dimensions and 16 items. Dimension 1, rejection, included items 1, 2, 3, 4, and 6; dimension 2, devaluation, included items 5, 7, 8, and 9; dimension 3, self-blame, included items 10, 11, 12, 13, 14, 15, and 16. It uses a five-point Likert scale, ranging from 1 (“Never”) to 5 (“Very Common”), with no reverse-scored items. The Cronbach’s α coefficient was 0.95. The total score on the scale ranged from 16 to 80 points, with higher scores indicating a greater degree of personal suicide stigma.

### Psychometric testing procedures

We contacted Professor Jurgita, the original author of the scale, through email to explain the research purpose and significance. We obtained the author’s consent and authorization. Following the guidelines for cross-cultural adaptation processes recommended by the American Academy of Orthopedic Surgeons, which included translation, synthesis, back-translation, expert consultation, and pre-testing, we adapted the scale accordingly (21, 22).

To translate the scale into Chinese, the original scale was independently translated by two nursing masters (native Chinese speakers with international English language testing system scores of 6.5 and 7.0) and one doctor of psychology with overseas study experience (passed the College English Test Band 6). The researchers organized the three translators and members of the research team (a total of four, including two nursing master’s students who have passed the College English Test Band 6, one nursing PhD, and one associate professor of nursing). The three translated drafts were compared, and inconsistent items were thoroughly discussed, revised, and integrated to preliminarily form Chinese translation version A. Subsequently, two additional individuals who have not been previously exposed to the original scale (one nursing faculty member from a university who has passed the College English Test Band 8, and one clinical nursing PhD who has passed the College English Test Band 6) were invited to independently back-translate the Chinese translated version A. A comparative analysis and adjustment of discrepancies were conducted with the researcher, and a preliminary back-translated version B was developed upon reaching a consensus. The back-translated version B was compared with the original scale regarding content, semantics, and other aspects to form the Chinese version B.

We designed an expert consultation letter to invite a total of eight experts, through either on-site distribution or email, to conduct a cross-cultural adaptation of the Chinese version B scale formed. They evaluated the clarity of its content, suitability of language, and potential ambiguities in item expression. The eight experts included one chief physician of clinical psychology, three associate chief physicians of clinical psychology, two associate professors of nursing psychology, one suicide crisis intervention expert, and one mental health specialist. Among them, two individuals had doctoral degrees and six had master’s degrees; two held senior professional titles, while six held associate professional titles. The age of the experts was 47.625 ± 7.347 years, with a working experience of 26.875 ± 10.696 years. The research team organized and discussed the expert consultation opinions, forming the next round of expert consultation questionnaires. The interval between each round of inquiry was more than 2 weeks until a consensus was reached among the experts, forming Chinese version C. A total of two rounds of expert consultations were conducted in this study.

A pre-survey was conducted on 40 adolescents with a history of suicide attempts using the Chinese version of scale C to assess the clarity and comprehensibility of each item in the scale and to determine if any additional content was necessary. The researcher carefully observed the subjects’ reactions to the items on the scale, inquired about the patient’s understanding of each item, and solicited their suggestions for improvement. Consequently, no modifications were needed after the pre-survey, and the formal Chinese version of PSSQ was established.

### Data collection

After obtaining approval from the supervisory department and relevant departments of the data collection hospital, the data collection personnel strictly screened the research subjects according to the inclusion and exclusion criteria. They explained the purpose and significance of the study to the subjects in person and obtained their consent before having them sign the paper version of the informed consent form. Subsequently, they scanned the QR code to fill out and submit the electronic questionnaire. All items were mandatory, and the questionnaire was filled out anonymously (to ensure data quality and test–retest reliability, the questionnaire included a field for participants to provide their hospital admission numbers). During the completion process, any additional questions were addressed in person. The estimated time for questionnaire completion was approximately 10–15 min. After completion, the researcher personally inspected the questionnaire submission status. Following the conclusion of each survey, the researcher reviewed the retrieved backend data on the same day, excluding questionnaires with evident logical errors or those where most items were answered with the same option. Additionally, to ensure test–retest reliability, 40 cases of the same study subjects, who were stable in their condition and willing to cooperate, were selected for a retest using the same questionnaire 2 weeks after the initial data collection. A total of 445 questionnaires were collected in this study, with 440 valid questionnaires, resulting in an effective response rate of 98.8%.

### Data analysis instruments

The valid data collected were cross-checked by two individuals and then downloaded into Microsoft Excel. Statistical analysis was conducted using IBM Statistical Package for the Social Sciences (SPSS) statistics (version 24.0) and IBM SPSS AMOS (version 26.0) software. Descriptive statistics such as mean ± standard deviation were used to describe normally distributed quantitative data, while median and quartiles were used to describe non-normally distributed quantitative data. Frequency and percentage were used to describe categorical data. Item analysis was conducted using the critical ratio method and correlation coefficient. The validity of the scale was assessed through content validity, construct validity, convergent validity, and discriminant validity. The questionnaire was randomly divided into two groups. In sample 1 (*n* ^1 =^ 220), EFA was conducted, and the suitability for EFA exploration was determined based on the Kaiser–Meyer–Olkin (KMO) measure and Bartlett’s sphericity test. In sample 2 (*n* ^2 =^ 220), CFA was performed, and the model fit and adequacy were evaluated using the comparative fit index (CFI), goodness of fit index (GFI), incremental fit index (IFI), Tucker–Lewis index (TLI), root mean square error of approximation (RMSEA), normed fit index (NFI), and χ^2^/df ratio. Acceptable model fit criteria included (23) CFI > 0.9, GFI > 0.9, IFI > 0.9, TLI > 0.9, NFI > 0.9, RMSEA < 0.08, and χ^2^/df ≤ 3. Reliability was assessed using Cronbach’s α coefficient, test–retest reliability, and half-split reliability. Statistical significance was determined at *p* < 0.05 for differences.

## Results

### Participant characteristics

A total of 440 adolescent patients who attempted suicide completed the survey, with a mean age of 10–19 years (15.757 ± 1.728). Other general information is detailed in **Table 1**.

**Table 1:** General information of the study participants (*n* = 440).

Project	*n*	%
Gender		
Male	75	17
Female	365	83
Residence		
Urban	275	62.5
Rural	165	37.5
School enrollment status		
Enrolled	129	29.3
On Hiatus	105	23.9
Intermittent schooling	208	46.8
Only child status		
Yes	175	39.8
No	265	60.2
Primary caregiver		
Father	50	11.4
Mother	196	44.5
Parents	137	31.1
Grandparents (paternal)	35	8.0
Grandparents (maternal)	10	2.3
Other	12	2.7
Parents’ marital status		
Married	328	74.5
Divorced	83	18.9
Separated	8	1.8
Other	21	4.8
Family history of mental illness		
Yes	49	11.1
No	391	88.9
Family history of suicide		
Yes	20	4.5
No	420	95.5
Doctor’s diagnosis results		
Depression	265	60.3
Anxiety disorder	34	7.7
Mood disorder	133	30.2
Obsessive–compulsive disorder	3	0.7
Schizophrenia	5	1.1
Frequency of suicide		
1	149	33.9
2	83	18.9
3	37	8.4
>3	171	38.9
Reasons of suicide		
Academic pressure or poor exam results	115	26.1
Family disputes or domestic violence	95	21.6
Love issues or emotional distress	44	10.0
All of the above	186	42.3
The most recent suicide time		
Three months ago	354	80.5
Six months ago	37	8.4
One year ago	49	11.1
Methods of suicide		
Jumping from height	58	13.2
Taking medication overdose	141	32.0
Wrist cutting	148	33.6
All of the above	93	21.1
Attitude towards death		
Fear	35	8.0
Willingness to contemplate death	405	92.0

### Item analysis

The project analysis results utilized the critical ratio method and correlation coefficient. The total scores of each item were ranked from highest to lowest. The high-score group comprised the top 27% (*n* = 119) of total scores, while the low-score group comprised the bottom 27% (*n* = 119) of total scores. Independent-sample *t*-tests were conducted to compare the mean differences in item scores between the two groups. The results revealed statistically significant differences between the high-score and low-score groups across 16 items (*t* = 9.571–20.430, *p* < 0.001) (24).

Pearson correlation analysis was conducted to examine the relationship between the scores of the 16 items and the total score of the scale. The results indicated that the correlation coefficient ranged from 0.478 to 0.725 (*p* < 0.001), indicating a moderate to high correlation. All original items were retained based on these results (19).

### Content validity

In this study, a total of eight experts were selected to conduct a content validity assessment of the Chinese version of the PSSQ. In the first round of expert consultations, the item-content validity index (I-CVI) exceeded 0.7 for all items, and the scale-content validity index (S-CVI) was 0.99. In the second round of expert consultations, the I-CVI remained above 0.7 for all items, and the S-CVI was 0.98. These results met the acceptable criteria for content validity (25), indicating that the Chinese version of the PSSQ exhibited good representativeness.

### Construct validity

In sample 1 (*n* ^1^ = 220), an EFA was conducted on 16 items, yielding a KMO measure of sampling adequacy of 0.906 and a significant Bartlett’s sphericity test (χ^2^ = 1,966.151; *p* < 0.001), indicating the appropriateness of factor analysis (26). Using principal component analysis and varimax rotation, three common factors with eigenvalues exceeding 1 were extracted, accounting for a cumulative variance of 64.621%. Two items (items 8 and 10) were deleted due to their factor loadings not aligning with the content dimensions of the items (26). A factor analysis was subsequently performed on the remaining 14 items, resulting in the extraction of three common factors. The cumulative variance contribution reached 66.729%, aligning with the predefined three dimensions of personal stigma related to suicide: rejection, devaluation, and self-blame. The factor loadings matrix after rotation for the Chinese version of the PSSQ is presented in **Table 2**.

**Table 2:** Factor loading matrix (*n* ^1^ = 220).

Item	Rejection	Minimization	Self-blame
1. I have been treated as less competent by others who learned about my suicidal thoughts or behavior.	**0.607**	0.260	0.324
2. I have been shunned or avoided when others found out about my suicidal thoughts or behavior.	**0.858**	0.202	0.114
3. Some people think that because of my suicidal thoughts or behavior, I will never be normal again.	**0.814**	0.160	0.211
4. My suicidal thoughts or behavior have led to some people treating me badly.	**0.812**	0.132	0.097
6. I have lost some valuable relationships after people found out about my suicidal thoughts or behavior.	**0.773**	0.196	0.152
5. People close to me do not want to talk about my suicidal thoughts or behavior and try to ignore them.	0.316	**0.658**	0.194
7. Other people ignore my suicidal thoughts or behavior, even though they know about them.	0.222	**0.753**	0.095
9. People try to minimize how serious my suicidal thoughts or behavior are.	0.128	**0.714**	0.166
11. Because of my suicidal thoughts or behavior, I doubt I will ever be normal again.	0.108	0.316	**0.693**
12. Because of my suicidal thoughts or behavior, I feel useless.	0.147	0.186	**0.852**
13. I blame myself for my suicidal thoughts or behavior.	0.295	−0.128	**0.616**
14. I feel less confident in my abilities because of my suicidal thoughts or behavior.	0.222	0.022	**0.850**
15. I feel like a burden to my family and friends because of my suicidal thoughts or behavior.	0.042	0.383	**0.758**
16. I feel like a failure because of my suicidal thoughts or behavior.	0.190	0.140	**0.863**
Eigenvalue	6.014	2.117	1.211
Variance contribution (%)	42.960	15.122	8.647
Cumulative variance contribution (%)	27.846	52.330	66.729

Items in bold black indicate factor loadings greater than 0.6, and thus these items are categorized under that dimension.

**Figure 1:**
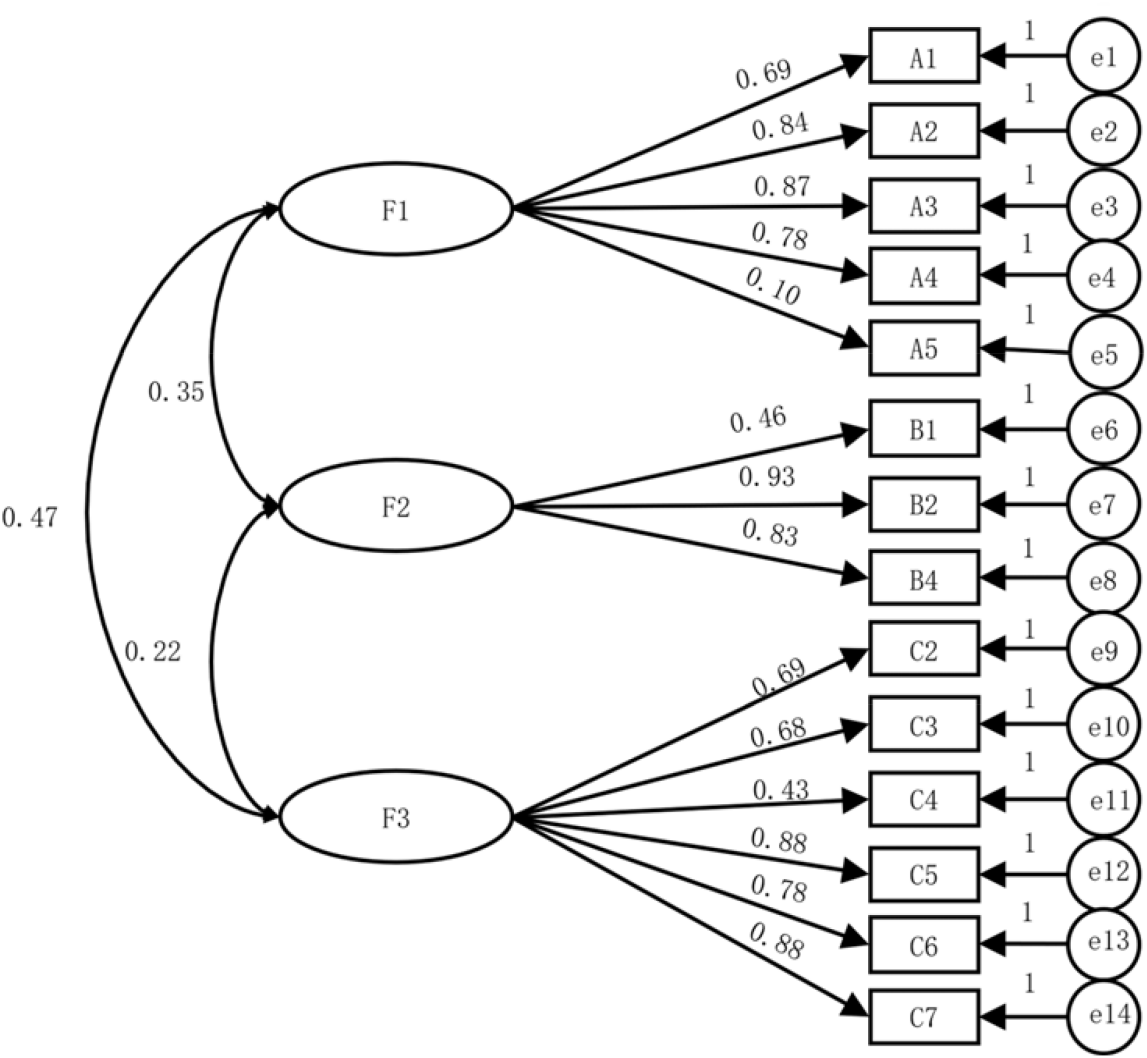
CFA model diagram.

Based on the CFA results, an initial structural equation model was established. Maximum likelihood estimation was used for parameter estimation, and the fit between the initial model and the data from sample 2 (n2 = 220) was evaluated (27). The fit indices for the initial model are shown in **Table 3**, and the model is analyzed in **Figure 1**.

**Table 3:** Fit indices of the model.

Statistical test statistic	Critical values for fit	Result values
**χ^2^/df**	≤3.000	2.186
**CFI**	>0.900	0.946
**GFI**	>0.900	0.912
**IFI**	>0.900	0.947
**TLI**	>0.900	0.934
**NFI**	>0.900	0.906
**RMSEA**	<0.08	0.074

### Convergent validity

For the Chinese version of PSSQ, the three latent variables—rejection, devaluation, and self-blame—demonstrated factor loadings exceeding 0.4 for each item, indicating good representativeness of the dimensions corresponding to each latent variable. The average variance extracted (AVE) for each dimension ranged from 0.511 to 0.603, while the composite reliability (CR) fell between 0.801 and 0.897 (26), suggesting satisfactory convergent validity of the Chinese version of PSSQ. Specific item factor loadings corresponding to each dimension are detailed in **Table 4**.

**Table 4:** Standardized regression coefficients for each item in the model.

**Item**	**Pathway**	**Dimension**	**Estimate**	**CR**	**AVE**
**A1**	<—	F1	0.691	0.814	0.511
**A2**	<—	F1	0.841		
**A3**	<—	F1	0.871		
**A4**	<—	F1	0.776		
**A5**	<—	F1	0.098		
**B1**	<—	F2	0.464	0.801	0.590
**B2**	<—	F2	0.930		
**B4**	<—	F2	0.831		
**C2**	<—	F3	0.688		
**C3**	<—	F3	0.885	0.897	0.603
**C4**	<—	F3	0.431		
**C5**	<—	F3	0.885		
**C6**	<—	F3	0.781		
**C7**	<—	F3	0.884		

### Discriminant validity

The rejection, derogation, and self-blame dimensions of the scale exhibited correlations ranging from 0.215 to 0.470 (*r* < 0.5, *p* < 0.05). The square roots of the AVE for each dimension ranged from 0.715 to 0.776, all exceeding the inter-dimensional correlation coefficients, indicating a certain level of correlation and discriminant validity among the dimensions (26). This suggested that the Chinese version of the PSSQ demonstrated ideal discriminant validity, as detailed in **Table 5**.

**Table 5:** Analysis of discriminant validity results.

	Rejection	Minimization	Self-blame
**Rejection**	0.511		
**Minimization**	0.216*	0.590	
**Self-blame**	0.470*	0.346*	0.603
**The square root of AVE**	0.715	0.768	0.776

*indicates p < 0.05; diagonal represents the square root of the AVE values.

### Reliability analysis

The internal consistency of the scale and its three dimensions was examined using Cronbach’s α coefficient, test–retest reliability, and half-split reliability. As depicted in **Table 6**, the Chinese version of the PSSQ demonstrated good reliability.

**Table 6:** Reliability test results.

Item	Cronbach’s α	Test–retest reliability	Half-split reliability
**Rejection**	0.831	0.898	0.788
**Minimization**	0.722	0.703	0.733
**Self-blame**	0.886	0.871	0.888
**PSSQ**	0.880	0.862	0.681

## Discussion

This study marked the first translation of the PSSQ scale into simplified Chinese, with subsequent examinations of its reliability and validity. Throughout the process of forming the Chinese version of the PSSQ, we rigorously adhered to established protocols. The examination results indicated that the scale exhibited robust content validity, construct validity, and reliability. All participants reported ease of comprehension regarding the scale’s items. Therefore, it stands as a reliable and effective tool for assessing the level of personal stigma among Chinese adolescents who have attempted suicide.

The Chinese version of PSSQ demonstrates clear and easily comprehensible item descriptions with good operability. Results from two rounds of expert consultations indicate an I-CVI exceeding 0.70 and an S-CVI exceeding 0.90, suggesting that each item accurately assesses the degree of personal stigma among adolescent suicide attempters. After EFA, apart from the removal of items 8 and 10 due to their inconsistent factor loadings with the original scale, no other differences were observed. This may be attributed to the strong desire for secrecy among suicide attempters influenced by traditional Chinese culture (28). Additionally, the study focused on adolescents whose understanding of suicidal behavior and stigma may differ (29, 30). Moreover, in China, research on personal stigma associated with suicide is indistinct from stigma related to mental illness, which could affect patients’ differentiation between internalized stigma associated with suicide and stigma associated with mental illness (16). A comparison of the three-factor model’s CFA results with commonly used measurement standards indicates a good fit for the three-factor model. Convergent validity assessment yielded AVE values ranging from 0.511 to 0.603, with all CR values exceeding 0.8. Furthermore, discriminant validity analysis based on Fornell–Larcker criteria for the Chinese version of the PSSQ demonstrated that the square roots of AVE for each dimension were at a minimum of 0.715, meeting the Fornell–Larcker criteria for discriminant validity (23). The Chinese version of the PSSQ scale demonstrates excellent psychometric properties, enabling accurate and reliable assessment of stigma levels among adolescent suicide attempters. This enriches the research landscape concerning the personal stigma associated with suicide in China.

### Limitations

This study has several methodological limitations. First, because of the lack of widely accepted standardized measures in the field of suicide in China, the study was unable to conduct criterion validation, thus restricting a comprehensive assessment of scale validity. Second, the lower number of male participants compared to female participants may reduce the generalizability of the scale. Future research should be aimed at improving the balance of gender representation in a wider and more diverse population. Furthermore, the sample collection in this study was limited to Zhejiang Province, which restricted the generalizability of the findings. Future research should expand the geographical distribution of samples to further validate the reliability and validity of the scale.

## Conclusion

The results of this study demonstrated that the Chinese version of the PSSQ exhibited good reliability and validity, with strong cultural adaptation to the Chinese context. In the future, this scale is poised to serve as a valuable tool in both research and clinical applications, particularly in assessing the perceived personal stigma of suicide among Chinese adolescent suicide attempters. Furthermore, the localization of this scale enriches the measurement tools for assessing suicide stigma perception in China, thereby contributing to the advancement of suicide research in areas including suicide prevention, mental health promotion, and the formulation of related public health policies. It is recommended for further exploration by domestic suicide researchers and validation and application in empirical studies.

In China, hospitals serve as the primary avenue for individuals who have attempted suicide to seek treatment (31). Healthcare professionals, as the primary sources of support for alleviating stigma-related stress and facilitating recovery after suicide attempts among adolescents, play a crucial role in assessing the personal stigma experienced by patients. This assessment helps clinical healthcare workers understand the current stigma status post-attempt and devise targeted measures to mitigate stigma-related stress, thereby promoting the recovery process. In the context of future healthcare practice in China, the assessment of stigma among adolescent suicide attempt survivors by healthcare professionals serves several key purposes: (1) to evaluate the current status of personal stigma experienced by adolescent suicide attempt survivors in China, (2) to explore the relationship between personal stigma among Chinese adolescent suicide attempt survivors and subsequent suicidal behaviors, and (3) to provide guidance for the development and evaluation of interventions targeting personal stigma among suicide attempt survivors and assess their effectiveness. This contributes to enhancing the awareness of medical staff and the whole of society about the current personal stigma experienced by suicide attempt survivors. Recognizing the importance of alleviating stigma for their recovery can help reduce the increased risk of subsequent suicide attempts caused by stigma and improve the quality of care in psychiatric wards.

## Data Availability

The original contributions presented in the study are included in the article/**Supplementary Material**. Further inquiries can be directed to the corresponding author.
