# Supplementary material for: Validation of the personal suicide stigma questionnaire among adolescents with suicide attempts in mainland China
Source: Front Psychiatry. 2024 Sep 13;15:1445247. doi: 10.3389/fpsyt.2024.1445247 (PMC11427941; doi:10.3389/fpsyt.2024.1445247)
Supplement: Supplementary file 1 [file DataSheet1.zip › DataSheet1/Inquiry Form(Chinese version).pdf]

「中文版 Personal Suicide Stigma Questionnaire——自杀个人污名量表 PSSQ」跨文化调适专家函询表

尊敬的专家：

您好！很荣幸能邀请您作为本课题研究的咨询专家，您丰富的经验和厚重的专业底蕴对本课题的顺利开展至关重要！非常感谢您在百忙之中对本课题的支持与指导。本研究旨在汉化、修订自杀个人污名量表（Personal Suicide Stigma Questionnaire，PSSQ），使其成为适合我国文化背景的、测量自杀者对个人自杀污名的评估工具。

PSSQ 量表是由澳大利亚维尔纽斯大学 Jurgita 教授和澳大利亚格里菲斯大学应用心理学院 John 教授，在焦点小组访谈 7 名有经验的临床医生和半结构式访谈 8 例自杀未遂患者的质性研究基础上，最终得到 16 个条目的多维度量表。该英文量表由 3 个维度组成，包括拒绝分量表（1-4、6）、贬低分量表（5、7-9）、自责分量表（10-16)，共 16 个条目。每个条目均采用 Likert5 级评分，从“从来没有”至“总是”分别计 1~5 分。

与精神疾病有关的污名对个人福祉会产生负面影响，该问题国内已展开了较多的研究。然而，自杀相关的污名却没有得到足够的关注。虽然自杀经常与精神疾病有关，但它本身不是精神疾病，因此需要进行单独的研究。污名使自杀者不愿寻求帮助，无法有效预防自杀后续行为，影响其重新融入正常生活。**故本研究通过汉化修订自杀个人污名量表，为了解自杀未遂患者对自杀污名的内化程度提供一种量化的工具，并在青少年自杀未遂人群中进行验证，构建出符合我国的自杀个人污名调查工具。**

本研究已取得原作者授权同意并通过 Brislin 双人翻译－回译法对 PSSQ 进行了翻译和回译，修改调整后初步形成了中文版 PSSQ。现邀请您对中文版问卷进行跨文化调试及内容效度的评价，使其更加具有实用性及科学性。感谢您对本课题给予的支持与帮助，期待您提出宝贵的意见和建议。**请您于 2024 年 1 月 17 日前将填写完成的专家咨询表发送至邮箱：** 2577272602@qq.com 如您在填写过程中，遇到任何疑惑，请随时与我或我的导师联系！祝您学术长青，万事胜意！

研究生：王晓宁

指导老师：缪群芳教授

联系电话：18834152794

第一部分 中文版自杀个人污名量表（PSSQ）

①请您仔细阅读问卷中的每个条目，对**条目内容与问卷的相关性**进行打分（**“1=不相关”、“2=弱相关”、“3=比较相关”、“4=非常相关”**）；

②**条目措辞是否清晰**，主要是指描述的清晰性、易理解性和复杂性是否合适（**“1=非常不同意”、“2=不同意，需要较大改进”、“3=同意，仍要较小改进”、“4=非常同意”**）直接点击☐即可自动打“√”。（若因 word 版本不兼容问题点击☐不能自动勾选，输入法输入“dagou”即可出现✔的符号。）如果您认为条目翻译内容需要修改以适应我国的特殊文化情况，请在修改意见一栏中给出意见。

序号	维度	原始问卷条目	翻译后问卷条目	条目内容				条目措辞				条目重要性					语言文化表达修正意见
				1	2	3	4	1	2	3	4	1	2	3	4	5	
	指导语	This questionnaire is about suicidal thoughts (wish to suicide and/or any plans to do so) and suicidal behaviour(attempting to take one’s own life in some way or harming oneself intentionally) and how other people react to them.<br>The questions below will ask you about experiences you might have had. There	该问卷旨在调查自杀人群的自杀想法（希望自杀和/或任何计划自杀）、自杀行为（试图以某种方式结束自己生命或故意伤害自己）以及周围人对他们自杀想法或行为的反应。<br>以下问题将会询问你是否有过相关经历，答案没有对错之分。你可能会发现，在下面所描述的情况中，你可能经历过很多，也可能很少有经历过。对于每个问题，请标出所描述事件发生的频率：从来没有 1					☐	☐	☐	☐						

		are no right or wrong answers. You might find that you have had experienced a lot of the situations described below or that you have experienced very few of them.For each question, please mark how frequently the described events have occurred: (1) never (2)seldom (3)sometimes (4)often (5)very often<br>how other people react to them	分；很少 2 分；有时 3 分；经常 4 分；总是 5 分<br>周围人对他们自杀想法或行为的反应													
1	**Rejection 拒绝**	I have been treated as less competent by others when they learned about my suicidal thoughts or behaviour	当别人知道我有过自杀的想法或行为时，他们认为我心理承受能力不足	☐	☐	☐	☐	☐	☐	☐	☐	☐	☐	☐	☐	☐	
2		I have been shunned or avoided when others found out about my suicidal thoughts or behaviour	当别人发现我有过自杀的想法或行为时，他们避免与我接触	☐	☐	☐	☐	☐	☐	☐	☐	☐	☐	☐	☐	☐	

3		Some people think that because of my suicidal thoughts or behaviour I will never be normal again.	由于我有过自杀的想法或行为，有人认为，我将永远无法恢复成正常人	☐	☐	☐	☐	☐	☐	☐	☐	☐	☐	☐	☐	☐	
4		My suicidal thoughts or behaviour have led to some people treating me really badly	由于我有过自杀的想法或行为，一些人对我的态度非常恶劣	☐	☐	☐	☐	☐	☐	☐	☐	☐	☐	☐	☐	☐	
5	**Minimization** 贬低	People close to me don’t want to talk about my suicidal thoughts or behaviour and try to ignore them.	我身边的人不愿意谈及我的自杀想法或行为，并试图忽视它们	☐	☐	☐	☐	☐	☐	☐	☐	☐	☐	☐	☐	☐	
6	**Rejection** 拒绝	I have lost some valuable relationships after people found out about my suicidal thoughts or behaviour.	在人们发现我有过自杀想法或行为后，与我的关系疏远了	☐	☐	☐	☐	☐	☐	☐	☐	☐	☐	☐	☐	☐	
7	**Minimization** 贬低	Other people ignore my suicidal thoughts or behaviour, even though they know about them.	即使其他人知道我有过自杀的想法或行为，他们也选择忽视它	☐	☐	☐	☐	☐	☐	☐	☐	☐	☐	☐	☐	☐	
8		Other people think I am just silly for thinking about suicide or atte	其他人认为我考虑自杀或试图自杀是很愚蠢的	☐	☐	☐	☐	☐	☐	☐	☐	☐	☐	☐	☐	☐	

		mpting suicide															
9		People try to minimise how serious my suicidal thoughts or behaviour are.	身边的人试图淡化我自杀想法或行为的严重性	☐	☐	☐	☐	☐	☐	☐	☐	☐	☐	☐	☐	☐	
10		Because of my suicidal thoughts or behaviour I feel nobody could admire me for anything I ever do	由于我有过自杀的想法或行为，我觉得我所做的任何事情都不可能得到别人的赞赏	☐	☐	☐	☐	☐	☐	☐	☐	☐	☐	☐	☐	☐	
11		Because of my suicidal thoughts or behaviour, I doubt I will ever be normal again.	由于我有过自杀的想法或行为，我怀疑自己是否还能够回到正常的状态	☐	☐	☐	☐	☐	☐	☐	☐	☐	☐	☐	☐	☐	
12	**Self-blame 自责**	Because of my suicidal thoughts or behaviour I feel useless	由于我有过自杀的想法或行为，我感到自己毫无用处	☐	☐	☐	☐	☐	☐	☐	☐	☐	☐	☐	☐	☐	
13		I blame myself for my suicidal thoughts or behaviour	我因自己有过自杀想法或行为而感到自责	☐	☐	☐	☐	☐	☐	☐	☐	☐	☐	☐	☐	☐	
14		I feel less confident in my abilities because of my suicidal thoughts or behaviour	由于我有过自杀的想法或行为，我对自己的能力感到不自信	☐	☐	☐	☐	☐	☐	☐	☐	☐	☐	☐	☐	☐	
15		I feel like a burden for my family and friends becaus	由于我有过自杀的想法或行为，我感到自己是家人和朋友	☐	☐	☐	☐	☐	☐	☐	☐	☐	☐	☐	☐	☐	

		e of my suicidal thoughts or behaviour	的负担														
16		I feel like a failure because of my suicidal thoughts or behaviour.	由于我有过自杀的想法或行为，我觉得自己是个失败者	☐	☐	☐	☐	☐	☐	☐	☐	☐	☐	☐	☐	☐	

第二部分 专家一般情况问卷

2.1 专家基本情况调查表					
姓名		性别		年龄（周岁）	
最高学历		职称		工龄（年）	
工作单位				职务	
研究领域及方向					
电子邮件				联系电话	

2.2 专家权威程度自评表			
以下内容请在相应栏内打“√”			
判断依据	依据程度(专家自我评价)		
	大	中	小
理论知识	☐	☐	☐
实践经验	☐	☐	☐
国内外动态研究	☐	☐	☐
直观感觉	☐	☐	☐

2.3 专家对填表内容的熟悉程度					
以下内容请在相应栏内打“√”					
熟悉程度	非常熟悉	较熟悉	一般	不太熟悉	不熟悉
专家自评	☐	☐	☐	☐	☐
