# Supplementary material for: Validation of the personal suicide stigma questionnaire among adolescents with suicide attempts in mainland China
Source: Front Psychiatry. 2024 Sep 13;15:1445247. doi: 10.3389/fpsyt.2024.1445247 (PMC11427941; doi:10.3389/fpsyt.2024.1445247)
Supplement: Supplementary file 1 [file DataSheet1.zip › DataSheet1/Inquiry Form(English version).pdf]

"Chinese version Personal Suicide Stigma Questionnaire —— Suicide Personal Stigma Scale PSSQ" cross-cultural adjustment expert letter inquiry form

Dear experts:

shalom! It is a great honor to invite you as a consulting expert of this project. Your rich experience and profound professional heritage are very important to the smooth development of this project! Thank you very much for taking time out of your busy schedule to support and give your guidance on this topic. The purpose of this study is to sinalize and revise the personal stigma Scale (Personal Suicide Stigma Questionnaire, PSSQ) to make it a suitable assessment tool to measure the stigma of personal suicide.

The PSSQ scale was based on a qualitative study by Professor Jurgita, Vilnius University, Australia and Professor John, School of Applied Psychology, Griffith University, Australia, in focus group interviews of seven experienced clinicians and semistructured interviews of eight attempted suicide patients to obtain a multidimensional scale of 16 items. The English scale consists of three dimensions, including the rejection subscale (1-4,6), the derogatory subscale (5,7-9), and the self-blame subscale (10-16), with a total of 16 items. Each entry was scored in Likert5, from 1 to 5 from "never" to "always".

The stigma related to mental illness negatively affects personal well-being, and more research has been conducted in the country. However, the suicide-related stigma has not received sufficient attention. Although suicide is often associated with mental illness, it is not psychiatric in itself and therefore requires separate studies. Stigma makes suicide reluctant from seeking help, unable to effectively prevent the follow-up of suicide and affect their reintegration into normal life. **Therefore, this study through the revision of suicide personal stigma scale in Chinese, to provide a quantitative tool to understand the internalization of suicide stigma of suicide attempt patients, and to verify in the adolescent suicide attempt population, to construct a personal stigma survey tool for suicide in China.**

In this study, the original author has approved and PSSQ has been translated and translated by Brislin two-person translation-back method. After modification, the Chinese version of PSSQ has been changed and formed. We hereby invite you to evaluate the cross-cultural debugging and content validity of the Chinese version of the questionnaire to make it more practical and scientific. Thank you for your support and help in this topic. I look forward to your valuable comments and

suggestions. Please send the completed expert consultation form to the email address: 2577272602 @ qq before January 17,2024.com If you have any doubts in the process of filling in, please feel free to contact me or my tutor! I wish you an evergreen academic experience and win everything!

Graduate student: Wang Xiaoning

Instructor: Professor Miao Qunfang

Contact number: 18834152794

Part I Chinese Version of the Suicide Personal Stigma Scale (PSSQ)

① Please read each item in the questionnaire carefully and score the correlation of the item content and the questionnaire ("1= irrelevant", "2= weak related", "3= more related", "4= very relevant");

☐☐② Whether the wording of the entry is clear, mainly refers to the clarity, understanding and complexity of the description ("1= very disagree", "2= do not agree, need greater improvement", "3= agree, still small improvement", "4= very agree") directly click to automatically hit "".(If it cannot be checked automatically due to word version incompatibility, the symbol of ✔ can appear by entering "dsign" in the input method.) If you think that the translation content of the entry needs to be modified to adapt to the special cultural situation of our country, please give your comments in the modification opinion column.

order number	dimension	Original questionnaire entry	Post-translated questionnaire entries	Item content				Entry wording				The importance of entry					Language and culture expression of amendments
				1	2	3	4	1	2	3	4	1	2	3	4	5	

**instruction**	This questionnaire is about suicidal thoughts (wish to suicide and/or any plans to do so) and suicidal behaviour(attempting to take one's own life in some way or harming oneself intentionally) and how other people react to them.<br><br>The questions below will ask you about experiences you might have had.There are no right or wrong answers.You might find that you have had experienced a lot of the situations described below or that you have experienced very few of them.For each question, please mark how frequently the described events	The questionnaire is designed to investigate suicidal thoughts (wish for suicide and / or any planned suicide), suicidal behavior (trying to somehow end their life or intentionally hurt themselves), and the reactions of people around them to their suicidal thoughts or behaviors.<br><br>The following questions will ask if you have had any relevant experience, with no right or wrong answers. You may find that you may or may have experienced much in the situations described below. For each question, mark the frequency of the described event: never 1; rarely 2; sometimes 3; often 4; always 5<br><br>Response of people around to		□	□	□	□		

		have occurred:(1) never (2)seldom (3)sometimes (4)often (5)very often how other people react to them	their suicidal thoughts or behavior														
1	**Rejection refuse**	I have been treated as less competent by others when they learned about my suicidal thoughts or behaviour	When people know that I have had suicidal thoughts or actions, they think that I am psychologically intolerant	☐	☐	☐	☐	☐	☐	☐	☐	☐	☐	☐	☐	☐	
2		I have been shunned or avoided when others found out about my suicidal thoughts or behaviour	When others find out that I have had suicidal thoughts or behavior, they avoid contact with me	☐	☐	☐	☐	☐	☐	☐	☐	☐	☐	☐	☐	☐	
3		Some people think that because of my s	Since I have had suicidal thoughts or action	☐	☐	☐	☐	☐	☐	☐	☐	☐	☐	☐	☐	☐	

		uicidal thoughts or behaviour I will never be normal again.	s, some people believe that I will never return to a normal person														
4		My suicidal thoughts or behaviour have led to some people treating me really badly	Since I have had suicidal thoughts or behavior, some people are very bad about me	☐	☐	☐	☐	☐	☐	☐	☐	☐	☐	☐	☐	☐	
5	**Minimization belittle**	People close to me don't want to talk about my suicidal thoughts or behaviour and try to ignore them.	People around me are reluctant to talk about my suicidal thoughts or actions and try to ignore them	☐	☐	☐	☐	☐	☐	☐	☐	☐	☐	☐	☐	☐	
6	**Rejection refuse**	I have lost some valuable relationships after people found out about my suicidal thoughts or behaviour.	Being anced from me after people discovered that I had suicidal thoughts or behaviors	☐	☐	☐	☐	☐	☐	☐	☐	☐	☐	☐	☐	☐	
7	**Minimization belittle**	Other people ignore my suicidal thoughts or behaviour, even though they know about them.	Even if others know that I have had suicidal thoughts or behaviors, they choose to ignore it	☐	☐	☐	☐	☐	☐	☐	☐	☐	☐	☐	☐	☐	
8		Other people think I am just silly for thinking about suicide or attempting suicide	Others think I am foolish to consider suicide or try to do it	☐	☐	☐	☐	☐	☐	☐	☐	☐	☐	☐	☐	☐	

9		People try to minimise how serious my suicidal thoughts or behaviour are.	People around me tried to downplay the seriousness of my suicidal thoughts or actions	☐	☐	☐	☐	☐	☐	☐	☐	☐	☐	☐	☐	☐	
10		Because of my suicidal thoughts or behaviour I feel nobody could admire me for anything I ever do	Since I have had suicidal thoughts or actions, I feel that nothing I do could be appreciated by others	☐	☐	☐	☐	☐	☐	☐	☐	☐	☐	☐	☐	☐	
11		Because of my suicidal thoughts or behaviour, I doubt I will ever be normal again.	Since I have had suicidal thoughts or actions, I doubt if I will be able to go back to a normal state	☐	☐	☐	☐	☐	☐	☐	☐	☐	☐	☐	☐	☐	
12	**Self-blame self-accusation**	Because of my suicidal thoughts or behaviour I feel useless	Because I have had suicidal thoughts or actions, I feel useless	☐	☐	☐	☐	☐	☐	☐	☐	☐	☐	☐	☐	☐	
13		I blame myself for my suicidal thoughts or behaviour	I blame myself for having had suicidal thoughts or actions	☐	☐	☐	☐	☐	☐	☐	☐	☐	☐	☐	☐	☐	
14		I feel less confident in my abilities because of my suicidal thoughts or behaviour	Since I have had suicidal thoughts or actions, I feel not confident in my abilities	☐	☐	☐	☐	☐	☐	☐	☐	☐	☐	☐	☐	☐	
15		I feel like a burden for my family and friends because of my suicidal thoughts or behaviour	With my suicidal thoughts or behaviors, I feel a burden to my family and friends	☐	☐	☐	☐	☐	☐	☐	☐	☐	☐	☐	☐	☐	

16		I feel like a failure because of my suicidal thoughts or behaviour.	Since I have had suicidal thoughts or actions, I feel like a loser	☐	☐	☐	☐	☐	☐	☐	☐	☐	☐	☐	☐	☐	

The second part is the expert general situation questionnaire

**2.1 Basic Information questionnaire of experts**					
surname and personal name		sex		Age (one year)	
highest education		professional ranks and titles		Length of Service (year)	
work unit				post	
Research field and direction					
E-mail				contact number	

**2.2 Self-evaluation form of expert authority degree**			
Please type the "√" in the corresponding column below			
Judgment basis	According to the degree of (expert self-evaluation)		
	big	centre	small
speculative knowledge	☐	☐	☐
hands-on	☐	☐	☐
Dynamic research at home and abroad	☐	☐	☐
Intuitive feeling	☐	☐	☐

**2.3 Experts' familiarity with the contents of the form**					
Please type the "√" in the corresponding column below					
degree of familiarity	Very familiar with	More familiar with	same as	Not familiar with	be unfamiliar with
Expert self-evaluation	☐	☐	☐	☐	☐
