# Supplementary material for: Validation of the personal suicide stigma questionnaire among adolescents with suicide attempts in mainland China
Source: Front Psychiatry. 2024 Sep 13;15:1445247. doi: 10.3389/fpsyt.2024.1445247 (PMC11427941; doi:10.3389/fpsyt.2024.1445247)
Supplement: Supplementary file 1 [file DataSheet1.zip › DataSheet1/Original Scale(English version).pdf]

PSSQ

This questionnaire is about suicidal thoughts (wish to suicide and/or any plans to do so) and suicidal behaviour (attempting to take one's own life in some way or harming oneself intentionally) and how other people react to them. The questions below will ask you about experiences you might have had. There are no right or wrong answers. You might find that you have had experienced a lot of the situations described below or that you have experienced very few of them.

For each question, please mark how frequently the described events have occurred: never (1), seldom (2), sometimes (3), often (4), very often (5).

	Never	Seldom	Sometimes	Often	Very often
1. I have been treated as less competent by others when they learned about my suicidal thoughts or behaviour.	1	2	3	4	5
2. I have been shunned or avoided when others found out about my suicidal thoughts or behaviour.	1	2	3	4	5
3. Some people think that because of my suicidal thoughts or behaviour I will never be normal again.	1	2	3	4	5
4. My suicidal thoughts or behaviour have led to some people treating me really badly.	1	2	3	4	5
5. People close to me don't want to talk about my suicidal thoughts or behaviour and try to ignore them.	1	2	3	4	5
6. I have lost some valuable relationships after people found out about my suicidal thoughts or behaviour.	1	2	3	4	5
7. Other people ignore my suicidal thoughts or behaviour, even though they know about them.	1	2	3	4	5
8. Other people think I am just silly for thinking about suicide or attempting suicide.	1	2	3	4	5
9. People try to minimise how serious my suicidal thoughts or behaviour are.	1	2	3	4	5
10. Because of my suicidal thoughts or behaviour I feel nobody could admire me for anything I ever do.	1	2	3	4	5
11. Because of my suicidal thoughts or behaviour, I doubt I will ever be normal again.	1	2	3	4	5
12. Because of my suicidal thoughts or behaviour I feel useless.	1	2	3	4	5
13. I blame myself for my suicidal thoughts or behaviour.	1	2	3	4	5
14. I feel less confident in my abilities because of my suicidal thoughts or behaviour.	1	2	3	4	5
15. I feel like a burden for my family and friends because of my suicidal thoughts or behaviour.	1	2	3	4	5
16. I feel like a failure because of my suicidal thoughts or behaviour.	1	2	3	4	5

Note: items 1-4,6 form the Rejection subscale, items 5,7-9 form the Minimization subscale, items 10-16 form Self-blame subscale. None of the items are reverse-scored.
